# Diff isomiRs: Large-scale detection of differential isomiRs for understanding non-coding regulated stress omics in plants

**DOI:** 10.1038/s41598-019-38932-w

**Published:** 2019-02-05

**Authors:** Kun Yang, Xiaopeng Wen, Suresh Mudunuri, G. P. Saradhi Varma, Gaurav Sablok

**Affiliations:** 1 Key Laboratory of Plant Resources Conservation and Germplasm Innovation in Mountainous Region (Guizhou University), Ministry of Education, Institute of Agro-bioengineering/College of Life Sciences, Guizhou University, Guiyang, 550025 Guizhou Province P. R. China; 2 Centre for Bioinformatics Research, SRKR Engineering College, Chinna Amiram, Bhimavaram, West Godavari District, Andhra Pradesh 534204 India; 3 Finnish Museum of Natural History, Helsinki, Finland; 4 Organismal and Evolutionary Biology (OEB) Research Programme, Department of Biological and Environmental Sciences, University of Helsinki, Helsinki, Finland

## Abstract

Plants have an amazing ability to cope with wide variety of stresses by regulating the expression of genes and thus by altering the physiological status. In the past few years, canonical microRNA variants (isomiRs) have been shown to play pivotal roles by acting as regulators of the transcriptional machinery. In the present research, we present Diff isomiRs, a web-based exploratory repository of differential isomiRs across 16 sequenced plant species representing a total of 433 datasets across 21 different stresses and 158 experimental states. Diff isomiRs provides the high-throughput detection of differential isomiRs using mapping-based and model-based differential analysis revealing a total of 16,157 and 2,028 differential isomiRs, respectively. Easy-to-use and web-based exploration of differential isomiRs provides several features such as browsing of the differential isomiRs according to stress or species, as well as association of the differential isomiRs to targets and plant endogenous target mimics (PeTMs). Diff isomiRs also provides the relationship between the canonical miRNAs, isomiRs and the miRNA-target interactions. This is the first web-based large-scale repository for browsing differential isomiRs and will facilitate better understanding of the regulatory role of the isomiRs with respect to the canonical microRNAs. Diff isomiRs can be accessed at: www.mcr.org.in/diffisomirs.

## Introduction

Functional genomics of abiotic stress tolerance in plants is at the forefront of the 21^st^ century. To improve plant longevity and sustainability, several approaches such as high-throughput expression profiling, next generation sequencing and emerging gene targeting are used in combination with each other and playing a key role in developing solutions^1–3^. In line with these approaches, multiple efforts have been leveraged to understand the transcriptional and post-transcriptional machinery, which includes the high-throughput profiling of the gene arrays and understanding the regulatory elements. Among the regulatory elements, non-coding RNAs, *e.g*. miRNAs^4,5^, artificial microRNAs (amiRNAs)^6^, circular RNAs (circRNAs)^7,8^, and long non-coding RNAs (lncRNAs)^9^ have been shown to be among the dominant class of non-coding RNAs in shaping the post-transcriptional events in plants. Plant microRNAs play a key role in defining the post-transcriptional regulation by altering the transcriptional regulation either through cleavage or translational suppression^10^. microRNA biogenesis has been long studied through the development of *HUA1 ENHANCER1* (*HEN*1) loss-of-function mutants, which is defective in methylating the microRNA duplex prior to exporting them to cytoplasm by Exportin5^11^. Biogenesis pathways of endogenous microRNAs are well established in plants elucidating the conversion from the pri-miRNAs to pre-miRNAs by Dicer-like (*DCL*1), followed by subsequent methylation by *HEN*1 methyltransferase and recruited by the ARGONAUTE 1 (*AGO1*) to form the RNA-induced silencing complex (*RISC*), which later causes the post-transcriptional suppression. miRNA biogenesis can be regulated by several factors such as whether the precursor is present in the introns. Stem-loop introns are often abbreviated as miRtrons and can affect the spliceosomal complex^12^. In concordant with the understanding of the microRNA’s biogenesis, relative understandings of their association to methylation^13,14^, their regulatory roles in abiotic stress, and identifying patterns of microRNA evolution have been long standing questions^15^, which are yet to be explored to understand the landscape of these 19–21 nt regulatory sequences. From the applicative point of view, microRNAs have been widely explored for transgenics, elucidating the developmental patterns^16^, designing artificial microRNA delivery to selectively regulate gene expression^17^ and to against plant virus^18^ and being used as immunomodulatory agents^19^.

Exploratory analyses of these microRNAs coupled with high-throughput discovery enabled the identification of another class of microRNAs called as isomiRs, which are canonical variants of microRNAs^20^. Along the timeline, much of the emphasis has been leveraged on the identification and classification of these isomiRs, leading to the establishment of three sub-types of isomiRs based on the substitutions and additions at the 5′- and 3′- terminus: 5′- isomiRs, 3′- isomiRs, and polymorphic isomiRs^21^. Although the 3′- terminal substitutions and additions contribute to the isomiRs diversity, 5′- terminal additions or substitutions are a major source of the diverse isomiRs, which can potentially lead to target site alterations, however this is yet to be widely explored among several plant species. Recently, it has been shown in plants that terminal modifications at the 3′- terminus are more evident with non-templated cytidine additions^22^. These previously observed terminal modifications were recently found in *Gossypium hirsutum* L. and soybean^23,24^ that were led by the conclusive role of uridylation as an important event to avoid the degradation and contribute to isomiRs biogenesis^25^. Concomitant with these reports, which establish 3′- uridylation as the dominant mechanism which widely correlates with the isomiR generation, target specificity of the isomiRs has also been revealed^26^. Although the role of the canonical miRNAs has been widely established in stress regulation^27–30^ and several repositories have developed to understand the wide role of miRNAs in stress and development^31^, recent efforts have revealed and reviewed the role of the isomiRs in plant stress^4,32,33^. Nevertheless, limited efforts were put into the identification of the isomiRs involving in stress response and the understanding of the shared and lineage specific role of isomiRs with respect to their parent miRNAs, which might be attributed to the lack of the high-throughput discovery of plant isomiRs and exploration on their subsequent role in the stress response and the developmental patterns. To support this, recent reports suggest the presence of the lineage specific expression and targets of miRNAs indicating that the previously reported miRNA’ome target abundance can be further increased by exploring the targets across the plant lineage^34^. Realizing the evolving context of the miRNAs and their potential targets, it is understandable that the diversity of the targets will also reflect the isomiR target diversity.

Sequencing and bioinformatics have accelerated the discovery of these isomiRs through the development of several tools such as SeqCluster^35^, miRSeqNovel^36^, isomiRID^37^, sRNAtoolbox^38^, isomiRex^39^, isomiRage^40^, DeAnnoIso^41^, isomiR2Function^42^. However, the application of these tools to understand stress regulation or the condition associated isomiRs is still undone. It is imperative to advance the understanding of the post-transcriptional machinery in stress regulation. Recent efforts by Zhang *et al*.^41^ provided a pre-compiled set of isomiRs across four plant species through the web-accessible isomiRBank. However, there are several limitations of isomiRBank such as: 1. isomiRBank presents isomiR identification for four species only and lacks the association of the identified isomiRs to corresponding stress. 2. isomiRBank doesn’t provides isomiR differential expression across stress conditions; 3. isomiRBank lacks the association of the differential isomiRs to targets and the association of the canonical miRNAs and isomiRs to PeTMs (endogenous target mimicry) respectively; 4. isomiRbank lacks the information on miRNA-isomiR target interactions.

Relative lack of these features prompted the development of the Diff isomiRs, which is the first web-based exploratory repository providing the differential isomiRs across 16 sequenced plant species, covering a total of 433 datasets and representing 21 different stresses and 158 experimental states. High-throughput analysis of the sRNA-Seq datasets revealed a total of 33,874 isomiRs and using the mapping-based and model-based methods allowed the detection of 16,157 and 2,028 templated differential isomiRs, respectively. Exploratory features such as detection of differential isomiRs according to species, stress and differential expression thresholds (log2FC and p-value) allows for ease of browsing differential isomiRs. A total of 2,010 differential isomiRs were found which are intersected to both embedded differential expression algorithms. To the best of the knowledge, this is the first integrated repository that provides the high-throughput detection of the differential isomiRs and features such as target prediction, log2FC across the stress conditions, mapping-based visualization, cross-accessibility of the isomiRs across the species and association of the canonical miRNAs and their templated isomiRs to PeTMs and also provides the miRNA-target interactions with respect to the isomiRs originating as a result of the terminal modification from the canonical miRNAs.

## Material and Methods

### Data resources

Genome datasets with corresponding features in GFF3 format, annotations and transcripts were downloaded for 16 plant species: *Arabidopsis thaliana, Brachypodium distachyon, Brassica rapa, Glycine max, Manihot esculenta, Medicago truncatula, Oryza sativa, Populus trichocarpa, Setaria italia, Solanum lycopersicum, Solanum tuberosum, Sorghum bicolor, Triticum aestivum, Vitis vinifera*,*Zea mays* from Phytozome version 11 accessible through www.phytozome.net/ ^43^ and *Hordeum vulgare* from Ensembl Genomes database accessible through ftp://ftp.ensemblgenomes.org/pub/plants/release-32/fasta/hordeum_vulgare/dna/Hordeum_vulgare.ASM32608v1.dna.toplevel.fa.gz ^44^. Small RNA datasets relevant to these species were identified through curated literature searches and can be found in Supplementary Table 1. For the aforementioned species, pre-miRNAs and mature miRNAs corresponding to these species were downloaded from miRBase version 21 accessible through http://www.mirbase.org ^45^ and plant microRNA database (PMRD) accessible through: bioinformatics.cau.edu.cn/PMRD^46^. rRNAs, tRNAs and snoRNAs were downloaded from RFAM (rfam.xfam.org/) and plant snoRNAs database (http://bioinf.scri.sari.ac.uk/cgi-bin/plant_snorna/home)^47^. Plant long non-coding RNAs were downloaded from CANTATAdb^48^, and GREENC^49^.

### Differential isomiR identification and development of Diff isomiRs

For the identification of differential isomiRs, all the smallRNA reads were firstly cleaned using a homemade Perl script which calls cutadapt for quality control and adapter trimming^50^ and the adapter-free reads were then collapsed and mapped to rRNAs, tRNAs, snoRNAs and long non-coding RNAs downloaded from abovementioned database. Reads mapped to any of the categories were excluded from further analysis. Filtered reads were mapped to genome features to estimate the proportion of the reads mapping to the relative genome features. The adapter-free, filtered and collapsed small RNAs were then mapped to pre-miRNAs allowing no mismatch using bowtie^51^. Templated isomiRs were defined as previously described in isomiR2Function^42^. For the estimation of the differential expression, we implemented two methods: 1. First method calls the differential expression of the isomiRs using the dispersion as implemented in the package DESeq2^52^ and 2. The second method hereby is referred to as mapping method estimating the differential expression by considering the mapping counts. Briefly, the abundance of each isomiRs is normalized by the total read count and p-value was estimated using the previously defined statistical tests^53,54^ and winflat^53^.

For the estimation of the p-value, we evaluated all the pairs as defined in a given condition by comparing the control vs stress in a single stress experiment and by comparing the control vs (stress)_n_ in case of multiple stress conditions. In case of multiple stresses defined in a given experiment, we also compared the defined (stress)_n_ to (stress)_n-1_. Log2FC was then evaluated as defined above for the single stress and the multiple stress experiments and the statistical value was estimated as defined by Wang *et al*.^55^ and described below:

$$\begin{array}{rcl}{\rm{p}}({\rm{x}}|{\rm{y}}) & = & {(\frac{{N}_{2}}{{N}_{1}})}^{y}\cdot [\frac{({\rm{x}}+{\rm{y}})!}{x!\cdot y!\cdot {(1+\frac{{N}_{2}}{{N}_{2}})}^{x+y+1}}]\\ {\rm{p}} & = & \min \{\sum _{k=0}^{k\ll y}p(k|x),\,\sum _{k=y}^{\infty }p(k|x)\}\end{array}$$

In the above defined condition, N_1_ and N_2_ define the total number of clean reads in the control and stress in the case of single stress experiment and in the case of multiple stress experiment, N_1_ and N_2_ define the total number of clean reads for the respective comparison. For the identification of the targets, psRNAtarget was used, which is accessible through plantgrn.noble.org/psRNATarget/^56^. For the integration of the target mimics, microRNAs and corresponding target mimics were retrieved from PeTMbase (http://petmbase.org)^57^. For the identification of the miRNA-target interactions, target interactions with enogenous RNAs were downloaded from PCeRBase available from http://bis.zju.edu.cn/pcernadb/index.jsp ^58^. Diff isomiRs has been developed using the MySQL as a backend database and uses PHP as a front end. Diff isomiRs uses WOFF2 font for faster loading of the web-pages. Diff isomiRs is hosted on a 16 core Intel Xeon machine with Ubuntu as an operating system and allows for the rapid searches and easy-to-browse interface is equipped with several search options.

## Results and Discussion

### Diff isomiRs: front end to the isomiR biology

**Figure 1:**
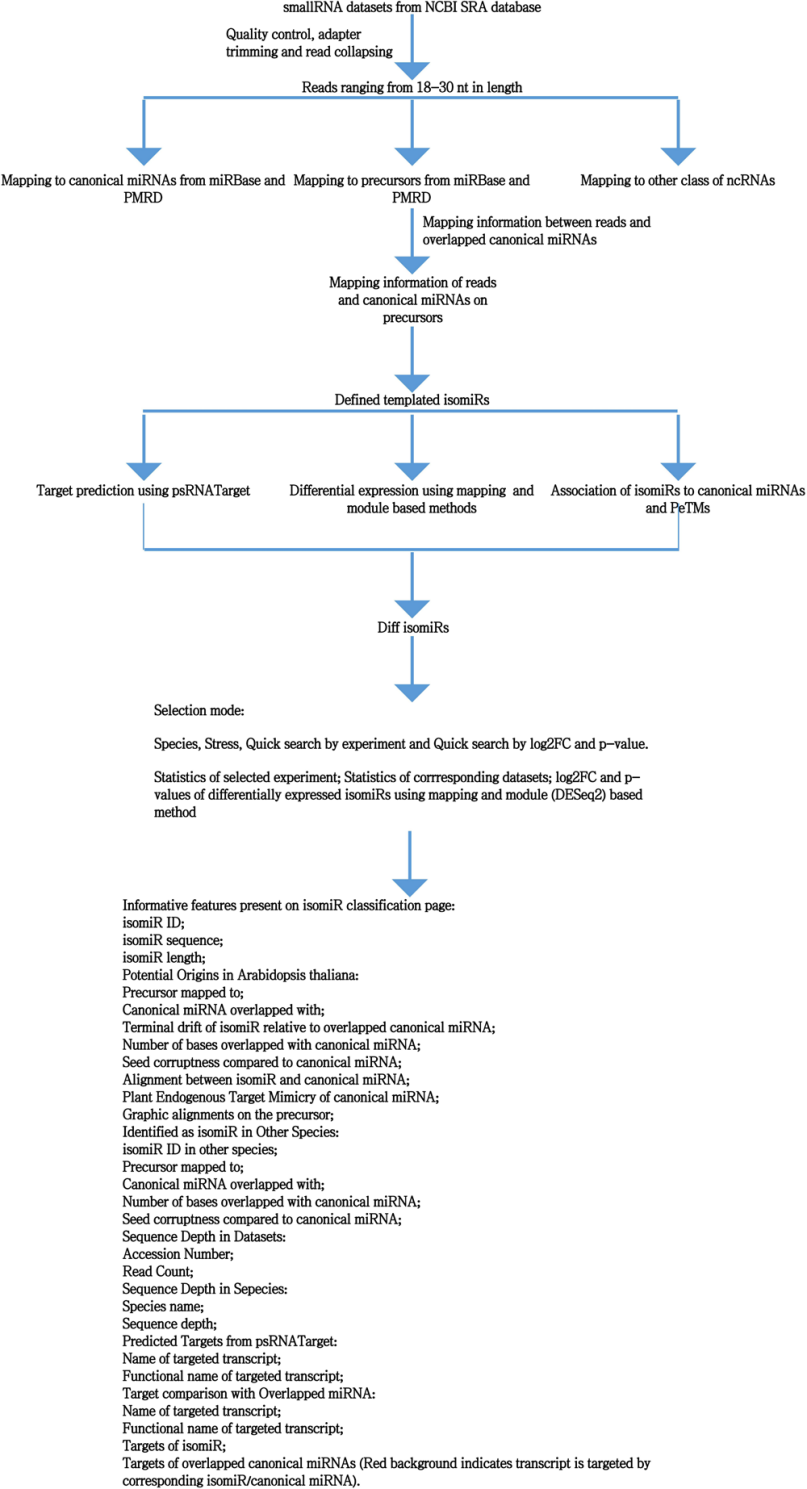
An overlay of the bioinformatics workflow in Diff isomiRs and the features in Diff isomiRs.

Post-transcriptional regulation is an important phenomenon controlled by several classes of regulatory RNAs, among which microRNAs play an important role as post-transcriptional regulators and as check points for transcriptional coordination. Leveraged by genome-wide microRNA profiling, substantial roles of microRNAs have been widely elucidated in development^10^ and stress regulation^29,59^. Limited evidences exist for the implication of isomiRs in stress, however it has been shown that stress widely regulates the expression of isomiRs and enhances the target repression repertoire^26,60^. To address this knowledge gap, we developed Diff isomiRs, which is an integrated information portal and catalogues the differential isomiRs in stress treatments across 16 plant species. The computational workflow along with the features implemented in the Diff isomiRs are presented in Fig. 1. Table 1 displays the corresponding number of experiments and datasets embedded in Diff isomiRs for each species. In total, Diff isomiRs contains 433 datasets representing 21 stress conditions classified into few types such as cold, heat, light, drought, submergence, salt etc. Although these canonical variants have been defined as ‘templated’ and ‘non-templated’ based on the subsequent processing of DROSHA/DICER enzymatic machinery^41^, Diff isomiRs provides detection and differential expression of templated isomiRs with two particular aims: 1. Robust identification of isomiRs with emphasis on the differential isomiRs patterns across the stress omics; and 2. Easy-to-explore interface of differential isomiRs and the canonical miRNAs to understand and reveal the prominent role of the nucleotide substitution events, which acts as check point for both the post-transcriptional controls and isomiRs biogenesis. For the identification of the differential expressed isomiRs, two differential expression algorithms provide a total of 16,157 and 2,028 differentially expressed isomiRs across the 21 different stress types implemented in Diff isomiRs. Table 2 displays the categorical distribution of the studied stress and the number of the differential isomiRs present in each stress according to the mapping-based method and model-based dispersion estimation. Interestingly, 2,010 differentially isomiRs were found across both algorithms. Supplementary Table 2 details the number of identified isomiRs in each representative stress for each species included in the Diff isomiRs.

**Table 1:** Summary of the species and the number of datasets used for each species respectively in Diff isomiRs.

Species	Datasets	Experiments
*Arabidopsis thaliana*	44	23
*Brachypodium distachyon*	23	14
*Brassica rapa*	32	8
*Glycine max*	56	19
*Hordeum vulgare*	6	3
*Manihot esculenta*	4	3
*Medicago truncatula*	16	3
*Oryza sativa*	50	23
*Populus trichocarpa*	2	1
*Setaria italica*	6	2
*Solanum lycopersicum*	22	5
*Solanum tuberosum*	2	1
*Sorghum bicolor*	8	5
*Triticum aestivum*	90	28
*Vitis vinifera*	2	1
*Zea mays*	70	19
Total	433	158

**Table 2:** Classification of the differential isomiRs across the studied stress in Diff isomiRs.

Stress	Mapping	DESeq2	Intersection
Alkalinity	623	3	2
Chitin	522	6	6
Cold	2,292	55	54
Drought	11,366	555	538
Drought + Salt	259	21	16
Flagellin	618	68	68
Graft	201	2	2
H2O2	238	13	13
Heat	6,797	807	777
Light	653	39	39
Mechanically treated	149	6	6
MeJA	154	2	2
Myc-lco	1,317	465	455
Nitrogen starvation	2,472	98	98
Nod	1,271	401	385
Osmotic	642	17	17
Phosphate starvation	1,998	420	399
Salt	4,530	209	199
Salt + submergence	690	20	20
Submergence	1,742	55	55
UV	765	89	85

Among the stress datasets, drought and salt stress revealed the most differentially expressed isomiRs with a total of 7,642 and 401 by mapping-based method and DESeq2 in drought stress and a total of 3,776 and 203 in salt stress respectively. Interestingly among these 397 isomiRs are represented by both methods in drought stress and 193 in salt stress respectively. Nevertheless, previous reports have widely established the role of the microRNAs in drought and salt stresses^61–64^, with limited reports addressing the role of isomiRs in stress which is mainly due to the lack of availability of high-throughput isomiR identification approaches. isomiR diversity as exemplified in above stress examples and present in Diff isomiRs can contribute to the better understanding of the post-transcriptional responses in these stresses. It is worth to mention that isomiR over expression has widely been documented^60^ and combination of the miRNAs and their canonical variants has been shown with improved target prediction^26^. Interestingly, we identified several important classes of miRNAs giving the most abundant isomiRs in each species (Table 3) such as ath-miRf10564-npr, which is similar to ath-miR172b-3p and targets mRNAs of AP2 proteins and plays an important role in development^65^. Similarly, in case of *Brachypodium distachyon*, we identified most abundant isomiRs originating from miR156 family which targets *SQUAMOSA PROMOTER BINDING PROTEIN* (SBP) genes and plays an important role in the developmental processes^66^.

**Table 3:** Table showing the most abundant isomiR in each species.

Species	isomiR	Read Count	Overlapped Canonical miRNAs	Source
*Arabidopsis thaliana*	t-ath-isomiR-563	3,195,620	ath-miRf10564-npr	PMRD
*Brachypodium distachyon*	t-bdi-isomiR-1994	1,927,304	bdi-miR156g-5p; bdi-miR156c	miRBase
*Brassica rapa*	t-bra-isomiR-124	25,196	bra-miR158-3p	miRBase
*Glycine max*	t-gma-isomiR-2697	100,778,426	gma-miR3522	miRBase
*Hordeum vulgare*	t-hvu-isomiR-33	5,036	hvu-miR156a; hvu-miR156b	miRBase
*Manihot esculenta*	t-mes-isomiR-111	20,1547	mes-miR166h	miRBase
*Medicago truncatula*	t-mtr-isomiR-1021	57,076	mtr-miR166a; mtr-miR166b; mtr-miR166c; mtr-miR166f; mtr-miR166g-3p	miRBase
*Oryza sativa*	t-osa-isomiR-5610	8,913,180	osa-miR156d; osa-miR156f-5p; osa-miR156j-5p	miRBase
*Populus trichocarpa*	t-ptc-isomiR-281	155,285	ptc-miRf10985-akr	PMRD
*Setaria italica*	t-sit-isomiR-381	284,000	sit-miR05-npr	PMRD
*Solanum lycopersicum*	t-sly-isomiR-309	35,470	sly-miR5302b-5p	miRBase
*Solanum tuberosum*	t-stu-isomiR-139	17,425	stu-miR6027	miRBase
*Sorghum bicolor*	t-sbi-isomiR-552	8,256,155	sbi-miR5564a	miRBase
*Triticum aestivum*	t-tae-isomiR-1622	1,512,354	tae-miR9664-3p	miRBase
*Vitis vinifera*	t-vvi-isomiR-266	505,276	vvi-miR167b; vvi-miR167e	miRBase
*Zea mays*	t-zma-isomiR-710	8,225,272	zma-miR166b-3p; zma-miR166c-3p; zma-miR166d-3p; zma-miR166e; zma-miR166f; zma-miR166g-3p; zma-miR166h-3p; zma-miR166i-3p	miRBase

Terminal modification either at the 5′- or the 3′- terminus plays an important role in directing the microRNA movement especially 5′- terminal nucleotides, which have been widely shown to play an important role in *ARGONAUTE* sorting, for example, preferential uptake of the sRNAs having 5′ cytidine has shown to be widely beneficial for *AGO5*^67^. These terminal modifications have not only been effective in associating the microRNAs to the AGO complexes but also revealing a class of microRNAs variants (isomiRs). On such classical example is the presence of the terminal additions at the 5′ nucleotides in miR157, which alters the binding efficiency of the miR157 to *SPL* genes^68^ Across the implemented stresses in Diff isomiRs, a total of 33,874 isomiRs were detected, with most of the isomiRs revealing the uridine at the 5′- terminus (Table 4). Previously uridine at the 5′- terminus has been shown to be one of the dominant phenomena of palnt miRNAs and AGO1 recruited smallRNAs^67^, which supports the present finding and also suggests uridine at 5′- terminus contributes to the isomiR functionality. Among all stresses, highest amount of the 3′- terminal cytidine addition was seen across the control, drought, salt and cold conditions in order (Table 5). It was reported that non-templated cytidine addition had been shown to be a dominant mechanism for isomiR biogenesis^22^. In present work, we observed that cytidine addition is also a dominant mechanism for templated isomiR biogenesis.

**Table 4:** Number of identified isomiRs and their terminal bases across different conditions.

Stress	Identified	5′ terminus	3′ terminus
Alkalinity	1481	A:324;C:277;G:223;T:657;	A:444;C:349;G:317;T:371;
Chitin	2363	A:544;C:447;G:305;T:1,067;	A:579;C:656;G:418;T:710;
Cold	8237	A:2,234;C:1,175;G:1,498;T:3,330;	A:2,125;C:2,259;G:1,828;T:2,025;
Control	22888	A:6,539;C:3,549;G:4,004;T:8,796;	A:5,673;C:6,328;G:4,967;T:5,920;
Drought	16737	A:4,783;C:2,674;G:2,990;T:6,290;	A:3,984;C:4,688;G:3,614;T:4,451;
Drought + Salt	545	A:94;C:98;G:158;T:195;	A:95;C:222;G:109;T:119;
Flagellin	2264	A:522;C:433;G:280;T:1029;	A:547;C:634;G:403;T:680;
Graft	331	A:46;C:50;G:23;T:212;	A:71;C:100;G:61;T:99;
H2O2	2464	A:779;C:342;G:461;T:882;	A:651;C:602;G:630;T:581;
Heat	8319	A:2,581;C:1,336;G:1,335;T:3,067;	A:1,855;C:2,414;G:1,892;T:2,158;
Light	1555	A:496;C:264;G:232;T:563;	A:364;C:368;G:515;T:308;
Mechanically treated	296	A:48;C:49;G:35;T:164;	A:85;C:92;G:56;T:63;
MeJA	1399	A:375;C:158;G:296;T:570;	A:450;C:304;G:289;T:356;
Myc-lco	205	A:21;C:29;G:24;T:131;	A:59;C:102;G:10;T:34;
Nitrogen starvation	3472	A:846;C:615;G:482;T:1,529;	A:943;C:935;G:698;T:896;
Nod	187	A:18;C:25;G:24;T:120;	A:49;C:95;G:14;T:29;
Osmotic	1998	A:558;C:241;G:457;T:742;	A:592;C:487;G:405;T:514;
Phosphate starvation	8314	A:2,439;C:1,225;G:1,582;T:3,068;	A:1,919;C:2,498;G:1,622;T:2,275;
Salt	10968	A:3,258;C:1,580;G:1,870;T:4,260;	A:2,903;C:2,932;G:2,301;T:2,832;
Salt + submergence	1908	A:513;C:251;G:404;T:740;	A:550;C:539;G:340;T:479;
Submergence	3996	A:1,051;C:567;G:867;T:1,511;	A:1,070;C:1,117;G:810;T:999;
UV	1544	A:489;C:264;G:223;T:568;	A:368;C:356;G:509;T:311;

**Table 5:** Number of 3′ + 1 isomiRs and observed terminal modification across different conditions.

Stress	Identified	3′ terminus
Alkalinity	112	A:46;C:17;G:18;T:31;
Chitin	137	A:53;C:23;G:18;T:43;
Cold	458	A:135;C:102;G:59;T:162;
Control	1053	A:312;C:239;G:131;T:371;
Drought	759	A:220;C:175;G:94;T:270;
Drought + Salt	50	A:8;C:17;G:7;T:18;
Flagellin	137	A:53;C:24;G:18;T:42;
Graft	31	A:9;C:6;G:1;T:15;
H2O2	125	A:41;C:25;G:15;T:44;
Heat	337	A:95;C:77;G:46;T:119;
Light	60	A:12;C:9;G:20;T:19;
Mechanically treated	30	A:8;C:9;T:13;
MeJA	91	A:33;C:14;G:9;T:35;
Myc-lco	34	A:11;C:15;G:2;T:6;
Nitrogen starvation	211	A:69;C:44;G:31;T:67;
Nod	33	A:10;C:13;G:3;T:7;
Osmotic	106	A:39;C:17;G:11;T:39;
Phosphate starvation	385	A:109;C:93;G:46;T:137;
Salt	610	A:182;C:142;G:75;T:211;
Salt + submergence	116	A:45;C:20;G:9;T:42;
Submergence	230	A:70;C:48;G:21;T:91;
UV	60	A:14;C:9;G:18;T:19;

### Diff isomiRs: Implemented browsing patterns for differential isomiRs

**Figure 2:**
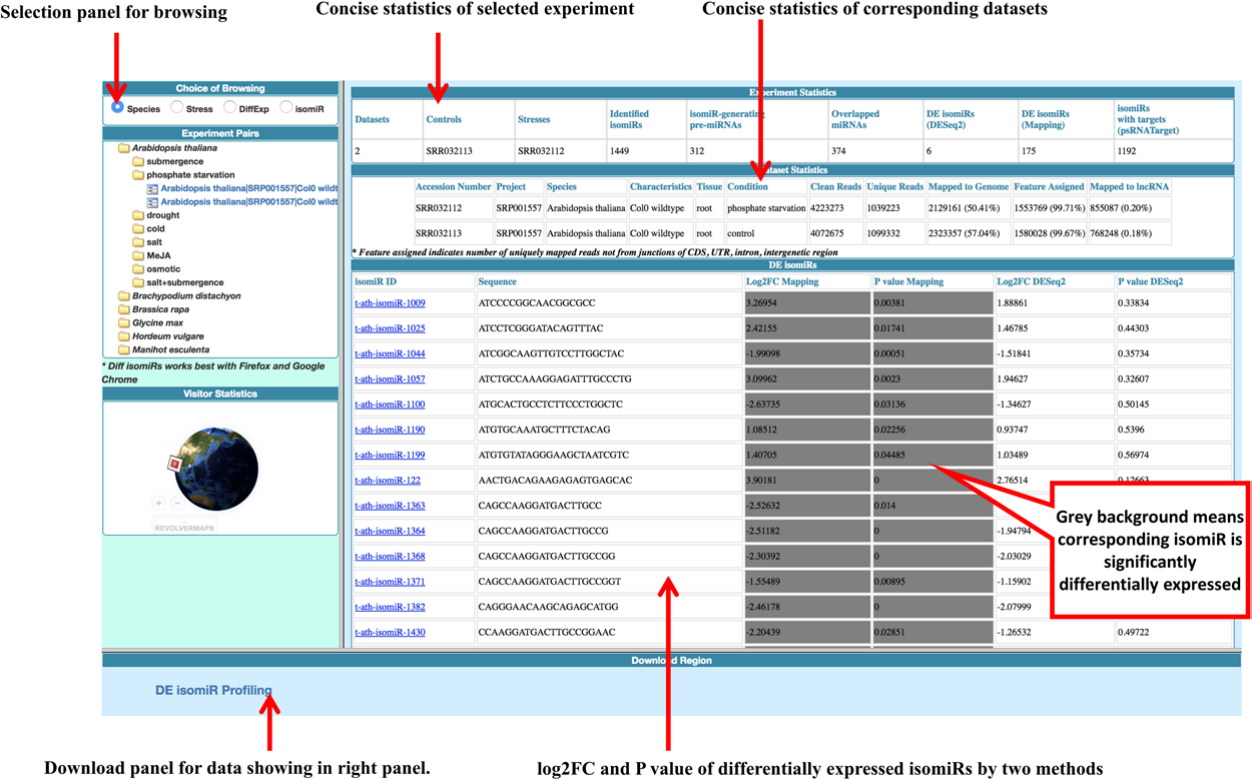
Browsing layout of Diff isomiRs showing the different selection steps viz. species, stress, isomiRs or differential expression.

Next generation sequencing has accelerated both the identification and characterization of isomiRs and the terminal modifications associated with isomiRs^65,69^. However, large-scale isomiR mining and development of open access exploratory portal are still lacking. Currently there is only a single repository, isomiRBank^41^ for plants, which provides pre-compiled set of isomiR across four plant species. However, isomiR diversity in stress is yet to be addressed. Considering this relative lack of open access web-based exploratory for stress associated isomiRs, Diff isomiRs was developed with an intuitive graphical display for ease of stress associated isomiR browsing. Diff isomiRs provides four features for mining of differential isomiRs (Fig. 2) such as selection according to species, stress, differential expression and experiment. Listed panel allows the quick selection of the species and the corresponding stress in Diff isomiRs. After clicking on experiments, the display page shows detailed features such as: 1. Number of identified isomiRs; 2. Number of pre- and mature-miRNAs used for isomiR identification; 3. Number of differentially expressed isomiRs as reported by two expression quantification methods; 4. Number of isomiRs with potential targets (Fig. 2). In addition to aforementioned experimental details, summary statistics such as: 1. Number of cleaned and collapsed reads; and 2. Reads assigned to genome and genome associated features and corresponding lncRNAs present in the respective species were shown at the same page (Fig. 2).

**Figure 3:**
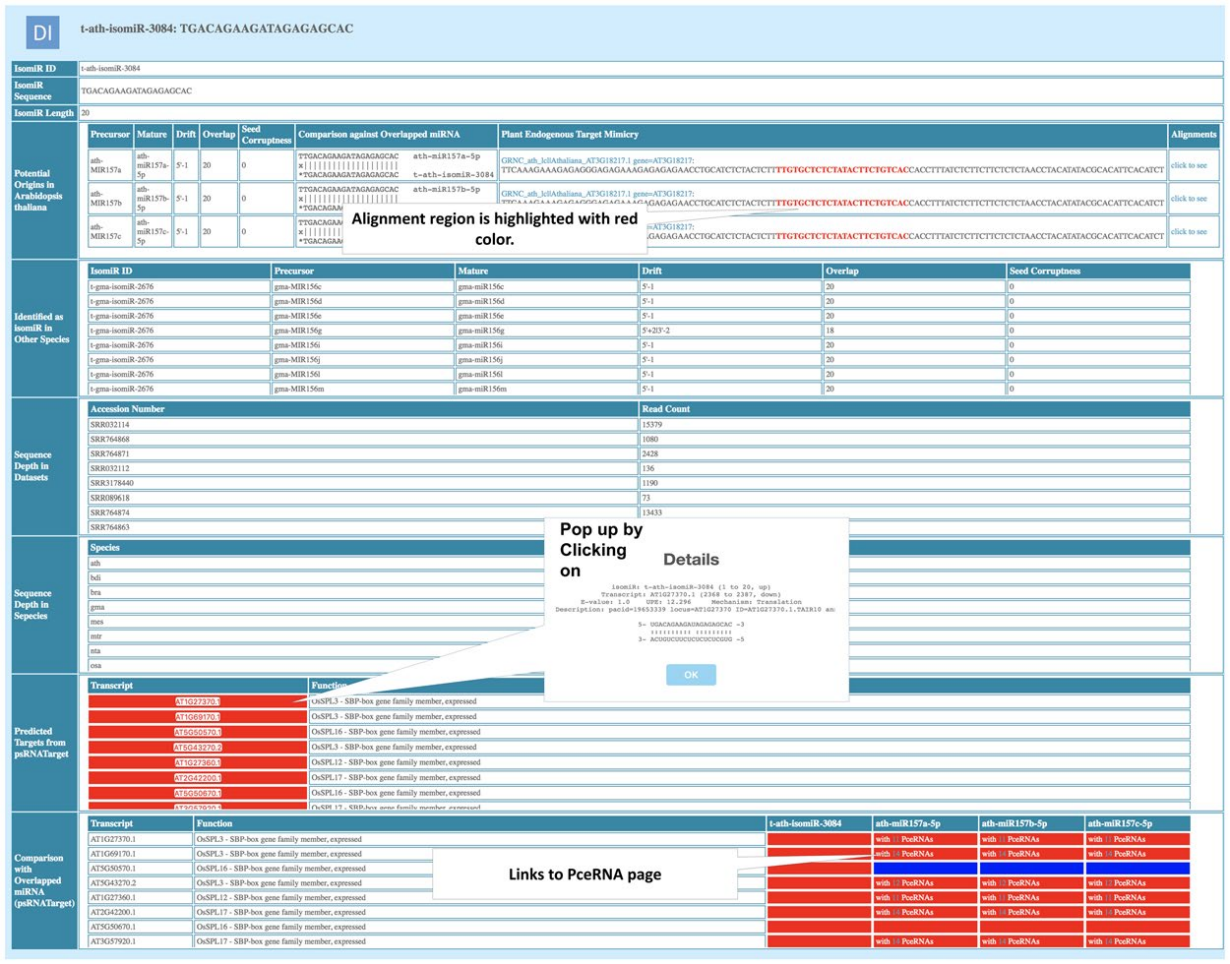
Differential isomiR specific page displaying features associated with the differential isomiRs such as pre-miRNAs, canonical mature miRNAs, SRA studies and their corresponding read counts, read depth in species, psRNAtarget predicted targets and corresponding function and alignment of the canonical miRNAs, isomiRs and the PeTMs with 50 bp window up- and down-stream of the endogenous mimic target.

To allow for the rapid visualization, differential isomiRs along with the log2FC and p-value calculated by mapping-based and model-based methods are listed in sorted tables along with sequence features. A quick download hyperlink allows to download the entire table in TAB-delimited format for manual curation (Fig. 2). Each differentially expressed isomiRs is further hyperlinked to isomiR specific page, which list several features, including: 1. Comparison of the mature miRNAs and isomiRs; 2. Read depth of the identified isomiRs across the datasets and their corresponding accession numbers; 3. Presence and absence of the isomiRs in other species; 4. Whether being identified as isomiR in other species; 5. psRNAtarget predicted target of the corresponding isomiRs along with the corresponding function and 6. Target comparison between isomiRs and corresponding canonical miRNAs (Fig. 3). To access the target site cleavage, each target is hyperlinked to a pop-up box, which displays the target site cleavage and the UPE and the corresponding coordinates of the target site (Fig. 3). Species, Stress, DiffExp and isomiR selection pages of Diff isomiRs allow for downloading of the information in TAB-delimited format, which can be easily exported for further manual curation. Target mimics in plants analogous to miRNA sponges in animals is a novel way to reduce the miRNAs targeting transcripts resulting in the overexpression of the target transcripts^70^. Recent approaches have enabled the high-throughput and computational-based prediction of plant endogenous target mimics (PeTMs, http://petmbase.org). The present version of the Diff isomiRs reports the alignment between the canonical miRNAs and isomiRs and the PeTMs with a 50 bp up- and down-stream window to understand the truncation events between the identified isomiRs, corresponding miRNAs and PeTMs (Fig. 3). Interestingly, we found a total of 4,230 isomiRs corresponding to 716 canonical miRNAs and 918 PeTMs from 11 species. Among these, species *Glycine max* represented the maximum number of isomiRs associated with PeTMs (962) and *Hordeum vulgare* represented the least number of isomiRs associated with PeTMs (7). In model plant *Arabidopsis thaliana*, multiple knock-down lines using artificial miRNA target mimicry have been previously reported^71^. Availability of these isomiRs associated with canonical miRNAs will accelerate the development of the multiple knock-down lines in model plants. In addition, Diff isomiRs can be widely used to establish the role of the isomiRs and can be further explored for understanding the truncation events, isomiRs biogenesis and miRNA target repository. miRNA-target interactions play an important role in revealing the targets, which can play a critical role in unravelling the temporal regulation of miRNAs. With the increased understanding of the miRNA-target interactions and their role in gene mediated pathways^72^, we downloaded all the miRNAs-target interactions from the recently published PCeRBase database^58^ and incorporate them into the Diff isomiRs for the canonical miRNAs and isomiRs for which the targets are predicted. Incorporation of these interactions provides an important feature to understand and expand the repertoire of endogenous interactions at the level of not only miRNAs but also with respect to isomiRs. isomiR specific page and corresponding target information are hyperlinked with the endogenous interactions as clickable links (Fig. 3). Availability of this information is critical to understand whether isomiRs can also play an important role in endogenous target interactions and for further validations through the easy-to-browse interface of Diff isomiRs which allows for the quick list of these interactions with respect to isomiR and canonical miRNAs.

### miRNA156 isomiRs: Case example of evolutionary conserved microRNAs in Diff isomiRs

Plant regulates its developmental processes through the systematic alterations of expressions of protein-coding genes. In addition to those, several non-coding RNAs especially miRNAs, which can alter the expression profile of the host genes, also play a critical role in regulating the host developmental processes. Vegetative phase is critical and crucial to the plant growth and development. Systematic expression of the genes along the leaf blades are required for the consecutive activation of the growth cascades. miRNA156 represents a classical example of the highly conserved microRNA family from the evolutionary point of view that not only on the basis of the target site conservation but also the evolutionary conservation among the sequence features that governs the target site identification and the formation of the fold back structures^73,74^. Complex regulatory events of miR156 have been widely observed ranging from the developmental functions to responding environmental stresses^75,76^. Recently, it has been shown that the miR156 regulated vegetative development is regulated and also in coordination with miR159, whose loss can temporally increase the miR156 level and hence increase the shoot development and delay vegetative growth^77^, although the conserved miR156 mediated inhibition of the *SQUAMOSA PROMOTER BINDING PROTEIN LIKE (SPL)*, which are critical regulators of the developmental transitions, has been well documented.

In particular, miR156 and miR157 both possess the ability to regulate the expression pattern of the major defining gene *SQUOMOSA PROMOTER BINDING PROTEIN-LIKE* (SPL). However, the binding efficiency of the miR156 and miR157 differs to SPL genes primarily due to the presence of the nucleotide additions at the 5′- terminus of the miR157 leading to imperfect binding^68^ and thus despite being more abundant than miR156, its mode of action is subsequently less as compared to miR156. The above example posed an interesting question suggesting the relative role of the nucleotide’s additions to the 5′- and 3′- end of the miRNAs and their relative binding efficiency. Gleaning from the above example, it is quite interesting to understand that whether these nucleotide additions in isomiRs of the parent miRNAs can affect the binding efficiency.

**Figure 4:**
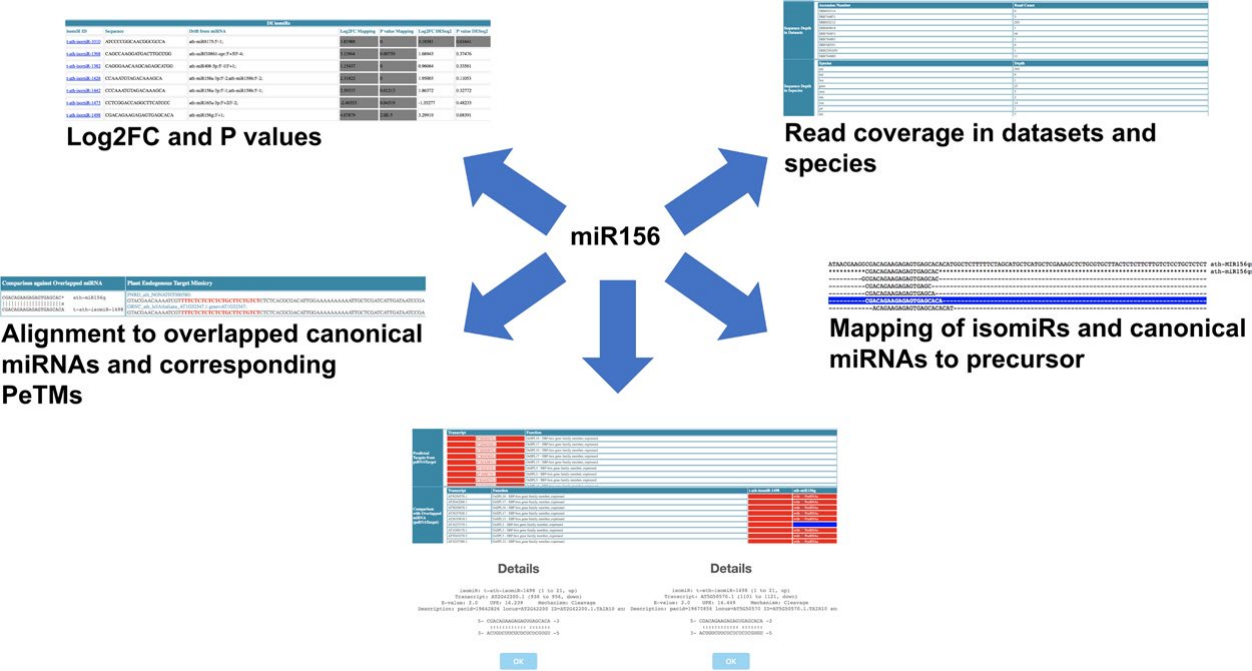
Case example of miR156 isomiRs in *Arabidopsis thaliana*.

**Figure 5:**
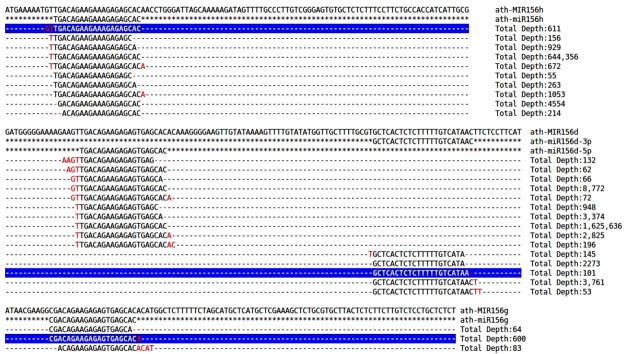
miR156 isomiRs and observed terminal modifications with read depth display in *Arabidopsis thaliana*.

However, recent studies by Wei *et al*.^76^ also highlights the role of the miR156 family in regulating the genes involved in carotenoid metabolism using sk156 mutant thus expanding the functional repertoire of miR156. In *Vitis vinifera*, miR156 plays an important role in fruit ripening and also downregulates the level of miR172, which targets *APETALA2 (AP2)* transcription factors^78^. Previous reports such as Baev *et al*.^60^ and Jeong *et al*.^65,69^ further elucidated the confirmatory higher expression of the isomiRs as compared to the canonical miRNA156 suggesting the evolutionary basis of isomiR biogenesis. To address, this question, there has been a relative lack of the studies providing a comprehensive set of the well characterized isomiRs showing the differential expression across stress conditions, which can be explored for the binding efficiency. Taking into account the critical role of miR156 as a complex regulator of the genes as well as regulator of other miRNAs with confirmatory isomiRs, we present a case browsing example of miR156 using *Arabidopsis thaliana* as an case example (Fig. 4), which highlights the relative features such as the identification of the differential isomiRs and their classification according to the log2FC and p-value, association of the predicted isomiRs with corresponding targets and association of the terminal modification with canonical miRNAs and corresponding endogenous target mimicry (PeTMs). Diff isomiRs detected a total of 107 isomiRs with 5′- and 3′- terminal modifications corresponding to 16 mature miRNAs in *Arabidopsis*. Among these, 209 differential isomiRs across 23 experiments and six across five were found by mapping-based and model-based methods, respectively. A total of 19 genes were predicted to be targeted by the miRNA156 in *Arabidopsis* revealing a total association with 5 endogenous target mimics (PeTMs). Since the classification of the isomiRs are defined by the terminal modifications at the 5′- and 3′- terminus, Fig. 5 shows the terminal modifications observed in the *Arabidopsis* miRNA156 along with the read depth display. These features with highlighted role of the terminal modifications will play a major role in understanding the role of terminal modification, which might be due to the *DCL1* imprecise cleavage or due to the post-transcriptional events. As per the Wei *et al*.^76^ miRNA156 network is not only limited to the *SPL* genes, therefore high-throughput discovery of these isomiRs will play a major role in improving target prediction and also to reveal the major complex patterns of the miRNA regulation.

Interesting, we observed a total of 162 targets specific to miR156 isomiRs using psRNATarget^56^ among which two of them were found to be AP2 transcription factors, which are targeted by miR172 family. Coordinated regulation of miR156 and miR172 class of miRNAs have been previously shown in *Vitis vinifera*^78^. Interestingly, among the targets, we observed AT1G67040.1 (TON1 RECRUITING MOTIF 22) and is targeted by miR838. miR838 has been previously shown to act as a negative feedback regulator of *DCL1* biogenesis^79,80^. A comprehensive list of the miR156 isomiR targets, which are identified in addition to previously described conserved SPL is provided (Supplementary Table 3). Recent efforts by Ahmed *et al*.^26^ pointed towards the advanced target prediction using the miRNAs and isomiRs in model plant *Arabidopsis thaliana*, which is well supported by the recent reports suggesting the miR1510, (legume clade miRNA) accumulation of the 22-nt isoform through mono-uridylation^81^, supported through the biochemical characterization revealing the mono-uridylation event as a result of HESO1^81^. Abundance of this isoform and its targeting mode of action in *Phaseoleae* species indicate that the isoform generation (isomiRs) from the parent miRNA is an evolutionary conserved phenomenon and fine tunes and enhances the target regulation. We believe that the swathing information on isomiRs and easy to search patterns in Diff isomiRs will play a significant role in increasing the knowledge base for isomiR biogenesis, alternative functions and expand evolutionary landscape of the isomiRs with respect to the parent miRNAs.

## Conclusion

Accelerated development of the sequencing technologies has currently been leveraged to understand the potential role of isomiRs and the functional impact of isomiRs as compared to the canonical miRNAs. Till now, stress associated isomiRs have not been widely explored due to the limited mining efforts for isomiRs across the stress datasets. Diff isomiR presents a next leap forward to understand the association of the isomiRs and their relative roles in stress by providing high-throughput mining of differential isomiRs across 16 plant species. The graphical web-based exploration not only allows for rapid assessment of isomiRs and associated features but also provides potential targets and the association of isomiRs, canonical miRNAs and target mimics. To conclude, this is the first web-based repository, which provides large scale isomiR discovery in stress and will significantly increase the understanding of templated isomiRs, their origin and potential roles of differential isomiRs in stress responses. An potent application of the DiffisomiRs is the exploration of the target mimics as previously observed and reviewed^82^ in conjuction with the corresponding miRNAs to increase the efficiency of the gene silencing approaches. To this end, a systematic in-depth identified isomiRs can provide a clue into the biochemical processes leading to the terminal modifications at small scale and thus in leading increasing the diversity of the smallRNAs’ ome.

## Supplementary information

Supplementary Table 1

Supplementary Table 2

Supplementary Table 3
